# Differences in socioeconomic and gender inequalities in tobacco smoking in Denmark and Sweden; a cross sectional comparison of the equity effect of different public health policies

**DOI:** 10.1186/1471-2458-10-9

**Published:** 2010-01-09

**Authors:** Frida Eek, Per-Olof Östergren, Finn Diderichsen, Niels K Rasmussen, Ingelise Andersen, Kontie Moussa, Mathias Grahn

**Affiliations:** 1 Department of Occupational and Environmental medicine, Lund University, Lund, Sweden; 2 Social Medicine and Global Health, Department of Clinical Sciences, Malmö, Lund University, Lund, Sweden; 3 Institute of Public Health Science, Department of Social Medicine, Copenhagen University, Centre for Health and Society, Øster Farimagsgade 5, DK-1399-Copenhagen K, Denmark; 4 National Institute of Public Health, University of Southern Denmark, Øster Farimagsgade 5, DK-1399 Copenhagen K, Denmark

## Abstract

**Background:**

Denmark and Sweden are considered to be countries of rather similar socio-political type, but public health policies and smoking habits differ considerably between the two neighbours. A study comparing mechanisms behind socioeconomic inequalities in tobacco smoking, could yield information regarding the impact of health policy and -promotion in the two countries.

**Methods:**

Cross-sectional comparisons of socioeconomic and gender differences in smoking behaviour among 6 995 Danish and 13 604 Swedish persons aged 18-80 years.

**Results:**

The prevalence of smoking was higher in Denmark compared to Sweden. The total attributable fraction (TAF) of low education regarding daily smoking was 36% for Danish men and 35% for Danish women, and 32% and 46%, respectively, for Swedish men and women. TAF of low education regarding continued smoking were 16.2% and 15.8% for Danish men and women, and 11.0% and 18.8% for Swedish men and women, respectively

The main finding of the study was that the socioeconomic patterning of smoking, based on level of education and expressed as the relative contribution to the total burden of smoking exposure, was rather different in Sweden and Denmark. Moreover, these differences were modified by gender and age. As a general pattern, socioeconomic differences in Sweden tended to contribute more to the total burden of this habit among women, especially in the younger age groups. In men, the patterns were much more similar between the two countries. Regarding continued smoking/unsuccessful quitting, the patterns were similar for women, but somewhat different for men. Here we found that socioeconomic differences contributed more to overall continued smoking in Danish men, especially in the middle-age and older age strata.

**Conclusion:**

The results imply that Swedish anti-smoking policy and/or implemented measures have been less effective in a health equity perspective among the younger generation of women, but more effective among men, compared to Danish policy implementation. The results also raises the more general issue regarding the possible need for a trade-off principle between overall population efficacy versus equity efficacy of anti-tobacco, as well as general public health policies and intervention strategies.

## Background

Socioeconomic inequalities in both morbidity and mortality have been found in virtually all studied countries in Europe including the Nordic countries [1,2]. It can be assumed that the socio-political context in a certain country, not only produces a pattern of general socioeconomic inequality (i.e. based on socioeconomic status, gender or ethnicity) but also determines a specific manner of interaction between such general inequality and health inequalities [3]. Moreover, it is likely that public health policy and health promotion interventions (a part of the socio-political context) play an important role in such a mechanism. This provides the rationale for performing international comparative studies of the specific mechanisms of health inequalities, in order to understand the impact of health policies and health promotion interventions on health and health equity in different countries.

Health behaviours, and the inequitable distribution of such determinants of population health, influence the future incidence of certain common chronic diseases and thus have a considerable impact on health status and utilization of health care services and costs. Estimates from WHO indicate that 37% of the burden of disease in Western Europe is attributable to tobacco smoking, alcohol consumption, diet and high cholesterol, physical inactivity and overweight [4]. Particularly tobacco smoking contributes to a large amount of the burden of disease in high income countries. Since this factor in most cases is distributed in a socioeconomically inequitable manner, it also contributes significantly to health inequalities in the mentioned part of the world [5].

Higher rates of both current and ever smoking have been found among lower educated men and women in north Europe. The educational differences were generally larger among those younger than 44 years, compared to older age groups. This age pattern suggests that socioeconomic differences in smoking-related diseases will increase in many European countries during the coming decades [6,7].

Furthermore, previous studies have shown that the prevalence of daily tobacco smoking is much higher in Denmark compared with neighbouring Sweden [8]. This despite the fact that both those countries are considered to be of rather similar socio-political type, i.e. both are considered typical "Nordic welfare states", with a similar general socio-political context [9]. However, some important elements in the welfare system differ between the two countries and the general economical development has also been different. E.g., the mobility on the labour market has been much greater in Denmark because of a radically different legislation with weak job security [9]. On the other hand, Denmark has experienced two decades of stable economical growth, with comparatively low unemployment figures, while Sweden experienced a deep crisis in the early 1990-ies with rapidly increasing unemployment, and then a considerable recovery towards the end of that decade [2]. However, the general level of ill health have remained stable in all the Nordic countries, at least until the mid-1990s, and the economic recession in Sweden was not reflected in any immediately deteriorating trend in population health or health inequalities. Lahelma et al [2] noted that, "in order to affect the population health and health inequalities in a notable way, the social changes have to be rather deep and dramatic" and further called for a broader examination of the social determinants of health inequalities [2], e.g. regarding the impact of country specific public health policy.

Considering this, it is interesting to note that there has been a strikingly different development of anti-tobacco policies in Sweden and Denmark [10]. In Sweden, government agencies have been much more active in promoting anti-smoking messages and implementing anti-tobacco policies (i.e. age limit for purchasing tobacco, smoking in public spaces, etc) while in Denmark such measures for a long time was considered to intrude on the integrity of the individual and only reluctantly implemented. It is therefore not surprising to find considerable overall differences in smoking prevalences in Sweden and Denmark, but to our knowledge the possible differences in the impact on socioeconomic patterns in smoking in the two countries has not been studied in detail, using data containing highly comparable, detailed information on the individual level from both countries.

We hypothesize that the mentioned differences, although within a framework of welfare policies considered to be largely similar and despite the absence of striking differences in overall health inequity measures, could result in socioeconomically different patterns in smoking in Sweden and Denmark.

Therefore, a study comparing mechanisms behind socioeconomic inequalities in tobacco smoking in the population of Denmark and Sweden, could potentially yield important general information regarding the interplay between socio-politically circumstances and implementation of health policy/promotion on the national level.

### Aim

The aim of this study was more specifically to compare the impact of educational level on the prevalence of daily smoking and the proportion of individuals who had successfully managed to quit smoking, in general representative samples of the Danish and the Swedish adult population in the Öresund region, as a way to assess general population efficacy as well as efficacy in an equity perspective.

## Methods

The data forming the study sample consist of subjects included in the health surveys conducted in Scania, Sweden and eastern Denmark 1999-2000. The two regions comprise large parts of Denmark and Sweden respectively. In Denmark, Copenhagen, the national capital is included in the studied area and it contains more than a third of the total population of Denmark. In Sweden, it is comprised of the region of Scania, which is one on the most densely populated area in the southernmost part of the country, holding one major city as well as rural areas and about 15% of the total population of Sweden. The national public health surveys of Denmark and Sweden has shown that the two regions show very similar patterns to the national ones, respectively, regarding population health and the major determinants of health (e.g. smoking) [11,12]. Therefore we consider the two chosen areas as reasonably representative of each country.

The primary aim of the data collection was to monitor the public health in the respective parts of the Öresund region in the context of the expected increase in cooperation, travel, migration and in general interaction as a consequence of the building of a bridge between DK and SE, and the entry of Sweden in EU in 1995. DK had already been member since 1973

### Study population

The total weighted study sample consist of men and women 18-80 years of age, randomly selected from the general populations of the Swedish county of Scania in southern Sweden and in the provinces of Zealand and Bornholm in eastern Denmark. For the year 2000 the Öresund region covers a total population of 3.5 million of which 1.13 million lives in Scania http://www.dst.dk/extranet/oresund1.

The total weighted sample consist of 20 092 persons. 249 persons (1.3%) did not respond to the smoking question; information about education was missing for 767 persons (3.8%).

#### The Swedish population

The Swedish data consist of 13 604 persons who responded to a postal public health survey in November 1999-April 2000. The questionnaire was sent by mail to a non-proportional age-, gender- and geographical area-stratified sample of 24 922 persons born between 1919 and 1981 and living in the 33 municipalities of the county of Scania. The final response rate was 58% of the net sample. The participants have in a previous study been shown to be representative for the total population of Scania, regarding age, gender and health care consumption [11].

#### The Danish population

The Danish sample consist of a subsample of the persons, who participated in the Health and Morbidity Survey (SUSY 2000 [12]) conducted by the Danish National Institute of Public Health. The data collection was made by means of personal interviews in three rounds in February, May and September 2000, respectively.

For the present analyses data from the subsample of initially 10 682 individuals 16 years or older in the counties and the Capital in Denmark east of Great Belt are included. Totally 7 473 persons participated, similar to a response rate for the region at 70%. Subjects younger than 18 years and older than 80 years were excluded in order to achieve the same age span in the Swedish and Danish data. The sampling probabilities in each of the counties were different in order to obtain county comparable sizes of the samples. The effective weighted Danish sample covered 6 995 subjects of whom 51% were women.

### The questionnaires

Both the Swedish and Danish surveys contained previously standardised/coordinated (i.e. between the two surveys) instruments and items regarding socio-demographic data (e.g. educational level), and information about health behaviours. The question about smoking read "Do you smoke?" with response alternatives "yes, daily", "Yes, but not daily", and "No". A following question asked (if No) "Have you ever smoked?" with response alternatives "Yes, quitted during last six months", "Yes, quitted more than six months ago" and "No". Put together, these questions formed four categories; (1) Current daily smoker, (2) Not smoker today, but have smoked previously, (3) Never smoked and (4) Current smoker, but not daily.

The Swedish survey was approved by the Regional Ethical Review Board in Lund (dnr 388/2004). The Danish survey was approved by "The Scientific Ethical Committee for Copenhagenand Frederiksberg Municipalities", on behalf of all local Scentific Ethical Committees in Denmark.

### Data management

#### Definition of variables

In the present study, two aspects of smoking status were examined. First, current daily smoking (*Daily smoker*) was used as outcome, in relation to the rest of the population (*Not daily smoker*). Secondly, all current smoking, daily and more occasionally, (*Current smoker*) was used as outcome in relation to those who did not smoke at the time, but had smoked previously (Quitters), hence excluding all never-smokers from the analyses.

Education was classified in four categories according to the International Standard Classification of Education (ISCED)-system (UNESCO 1997);(1) up to 10 years of education (ISCED level 0-2), (2) 11-12 years of education (ISCED level 3), (3) 13-14 years of education and (ISCED level 4-5) and (4) 15+ years of education (ISCED level 6). The highest education group (level 4, 15+ years) was used as reference group.

#### Statistical analyses

Analyses were performed stratified for country (Sweden or Denmark), gender and three age groups (18-44, 45-64, and 65+ years). A few different measures were calculated to illustrate the relation between smoking and education in the populations. Odds ratios (ORs) were calculated through logistic regression. To quantify the proportion of smokers among "exposed" (here less educated) people that could be attributed to the exposure (i. e low education), attributable fraction (AF) were calculated for each level of education according to the formula R_exp_-R_unexp_/R_exp_[13]. Prevalence was used as risk estimate ("R"). The AF can be interpreted as the proportion of smoking among the low educated that could be attributed to the low education, and that would be prevented if the low educated smoked to a similar extent as the highest educated. In order to also take into account distribution of educational level among the smokers, stratum specific total attributable fractions (**sTAF**) and **overall **total attributable fraction (TAF) were calculated [13]. The **sTAF was **obtained for each educational level by multiplying the AF for the level by the proportion of all cases (i. e. smokers) in the total population that were "exposed" to that educational level. The **sTAF **measure can be interpreted as the additional proportion of smoking in the population that can be attributed to each level of education compared with the highest one. **TAF **was obtained by summing the **sTAFs **from the three educational levels. The TAF can be interpreted as the total proportion of the smoking in the population that could be prevented if the low educated smoked at a similar extent as the highest educated. For the variable "current smoker vs quitter", all never-smokers were excluded from the analyses, why the interpretation of the **sTAF/TAF **becomes a little different. Instead of indicating the proportion of smoking in the population that could be attributed to low education, the **sTAF/TAF **for current smoking should be interpreted as the proportion *of continued smoking among all ever-smokers*, or "unsuccessful quitting", that could be prevented if the low educated would quit smoking to a similar extent as the high educated. All analyses and calculations were performed in SPSS 13.0 (SPSS Inc., 2004).

#### Weighting

Both populations were weighted to correspond to the current population in the geographical area from where the study population was drawn. The Swedish weighting was based on the variables geographical strata, age and gender, giving 360 weight groups (60 geographic strata × 3 age categories × 2 gender categories). Each group was given a weight corresponding to the proportion of that group in the total population in Scania.

The Danish data was weighted to be representative of the whole Danish population.

## Results

**Figure 1:**
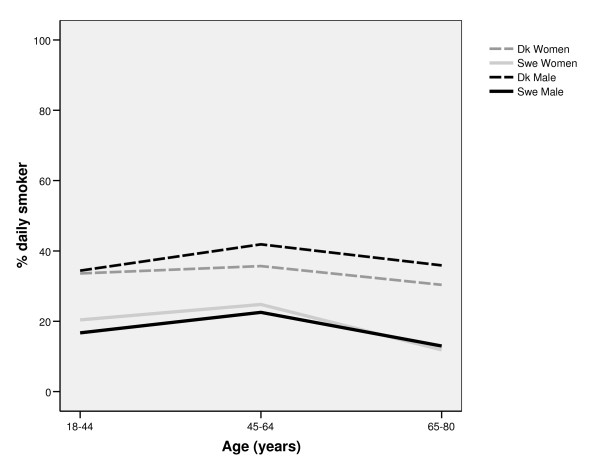
**Proportion of daily smoking in different age groups among Swedish and Danish men and women**.

**Figure 2:**
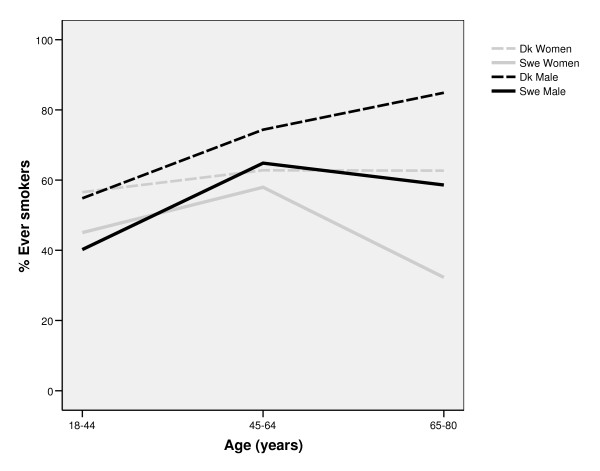
**Proportion of ever smoking in different age groups among Swedish and Danish men and women**.

**Figure 3:**
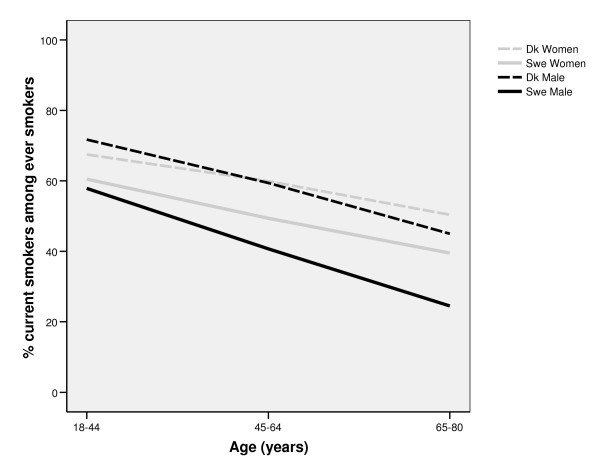
**Proportion of unsuccessful quitting (current smoking among ever smokers) in different age groups among Swedish and Danish men and women**.

Among all age groups, and among both men and women, daily smoking was more prevalent in Denmark than in Sweden. In both countries, smoking was most prevalent in the middle aged (45-64 years) groups (figure 1). In Denmark, smoking was more common among men than among women. The pattern was partly the opposite in Sweden; a larger share of the women than of the men were daily smokers in the youngest and middle aged group, while there was no gender difference in the oldest age group. Ever smoking was most prevalent among Danish men, in particular in the older age groups. There was a shift in gender inequalities in both Denmark and Sweden; in the youngest age group, ever smoking was more prevalent among women than among men, while ever smoking was much more common among men in both countries in the older age groups (figure 2). As expected, the proportion of current smokers among ever smokers was highest in the youngest age group, in both countries. The proportion of current smokers was higher in Denmark compared to Sweden, in all ages. The most successful quit-rates were found among Swedish men (Figure 3).

A larger share of the Danish men and women had the highest education (level 4), compared to Swedish men and women. The difference was most obvious in the oldest age group. In the youngest age group, it was slightly more common to have the lowest education (level 1) among Danish men compared to Swedish men. The prevalence of young women with the lowest education level was almost similar in Sweden and Denmark. In the middle- and oldest age groups, the lowest education level was much more common in Sweden compared to Denmark (table 1). The pattern was similar when looking at ever smokers only (table 2).

**Table 1:** Distribution of education level 1-4 among Swedish and Danish men and women (n = 19843)

		Women		Men	
		
		Denmark	Sweden	Denmark	Sweden
18-44 years		%	%	%	%
		n = 1586	n = 3169	n = 1599	n = 3220
	Education level				
	15+ years education (level 4)	30.1	21.6	26.6	20.2
	13-14 years education (level 3)	41.1	24.4	36.9	20.8
	11-12 years education (level 2)	16.1	41.1	19.9	46.0
	Up to 10 years education (level 1)	12.8	12.9	16.5	12.9
45-64 years					
		n = 1103	n = 2366	n = 1118	n = 2371
	Education level				
	15+ years education (level 4)	23.4	19.4	23.0	20.5
	13-14 years education (level 3)	28.7	17.9	25.2	16.8
	11-12 years education (level 2)	27.9	14.1	36.8	14.6
	Up to 10 years education (level 1)	20.0	48.6	15.1	48.1
65-80 years					
		n = 578	n = 1355	n = 505	n = 1124
	Education level				
	15+ years education (level 4)	10.3	5.7	21.0	12.3
	13-14 years education (level 3)	18.5	6.7	16.8	10.5
	11-12 years education (level 2)	30.6	7.5	35.8	7.2
	Up to 10 years education (level 1)	40.6	80.1	26.4	70.1
All ages					
		n = 3267	n = 6889	n = 3222	n = 6715
	Education level				
	15+ years education (level 4)	24.3	18.0	24.4	19.1
	13-14 years education (level 3)	32.9	19.0	29.6	17.8
	11-12 years education (level 2)	22.6	25.8	28.3	28.8
	Up to 10 years education (level 1)	20.2	37.2	17.6	34.3

**Table 2:** Distribution of education level 1-4 among ever smoking Swedish and Danish men and women (n = 10693)

		**Women**		**Men**	
		
		**Denmark**	**Sweden**	**Denmark**	**Sweden**
		
18-44 years		%	%	%	%
		
	Education level	n = 897	n = 1410	n = 875	n = 1281
	15+ years education (level 4)	23.1	15.7	21.7	14.9
	13-14 years education (level 3)	42.9	22.3	35.4	19.3
	11-12 years education (level 2)	17.3	42.3	21.4	45.3
	Up to 10 years education (level 1)	16.6	19.7	21.5	20.6
45-64 years					
					
	Education level	n = 690	n = 1353	n = 829	n = 1512
	15+ years education (level 4)	23.8	18.1	20.6	18.4
	13-14 years education (level 3)	28.8	17.9	24.7	16.6
	11-12 years education (level 2)	27.2	15.7	38.1	14.0
	up to 10 years education (level 1)	20.2	48.2	16.6	50.9
65-80 years					
					
	Education level	n = 361	n = 419	n = 428	n = 636
	15+ years education (level 4)	10.6	7.6	17.6	12.5
	13-14 years education (level 3)	18.1	8.8	16.2	13.1
	11-12 years education (level 2)	32.4	8.6	38.1	5.8
	up to 10 years education (level 1)	38.9	75.0	28.1	68.7
All ages					
					
	Education level	n = 1949	n = 3182	n = 2133	n = 3429
	15+ years education (level 4)	21.0	15.7	20.4	16.0
	13-14 years education (level 3)	33.3	18.8	27.4	17.0
	11-12 years education (level 2)	23.6	26.9	31.3	24.4
	up to 10 years education (level 1)	22.1	38.6	20.9	42.6

Never-smokers (n = 9150) excluded

The relation between education and smoking was clear in the two youngest age groups, with increasing likelihood of smoking among the groups with lower education (table 3). The one exception is among Swedish middle aged men, among which only the least educated group had significantly increased likelihood of daily smoking. Generally, the educational trend was strongest in the youngest age group, in both countries and among both men and women. In the oldest age group, the lower educated groups did not differ significantly from the highest education group regarding smoking, except among Danish men where the education level 1 and 2 had significantly increased odds ratios for daily smoking.

**Table 3:** Likelihood* of being a daily smoker by educational level, and attributable fractions, for men and women, Denmark and Sweden

		**Women**		**Men**	
		
		**Denmark**	**Sweden**	**Denmark**	**Sweden**
		
18-44 years					
					
	OR educational level 4	1	1	1	1
	OR educational level 3 (95% CI)	2.1 (1.6-2.8)	2.1 (1.5-2.9)	1.8 (1.4-2.4)	1.4 (1.0-2.0)
	OR educational level 2 (95% CI)	2.8 (2.0-4.0)	2.9 (2.1-3.9)	2.6 (1.9-3.7)	1.8 (1.4-2.5)
	OR educational level 1 (95% CI)	4.8 (3.4-6.9)	6.6 (4.7-9.2)	4.8 (3.4-6.7)	5.3 (3.8-7.5)
	Attributable fraction (AF) educational level 3	0.41	0.47	0.36	0.25
	Attributable fraction (AF) educational level 2	0.51	0.59	0.49	0.41
	Attributable fraction (AF) educational level 1	0.63	0.77	0.63	0.74
	sTAF educational level 3	0.17	0.10	0.12	0.04
	sTAF educational level 2	0.10	0.27	0.12	0.18
	sTAF educational level 1	0.13	0.19	0.17	0.21
	Total attributable fraction (TAF)	0.41	0.55	0.40	0.43
45-64 years					
					
	OR educational level 4	1	1	1	1
	OR educational level 3 (95% CI)	1.7 (1.2-2.5)	1.7 (1.2-2.4)	1.9 (1.4-2.8)	1.0 (0.7-1.4)
	OR educational level 2 (95% CI)	1.8 (1.2-2.5)	2.8 (2.0-4.0)	2.0 (1.4-2.8)	1.1 (0.8-1.6)
	OR educational level 1 (95% CI)	3.0 (2.0-4.4)	2.4 (1.8-3.2)	2.6 (1.7-3.9)	2.2 (1.7-2.9)
	Attributable fraction (AF) educational level 3	0.31	0.35	0.35	-0.03
	Attributable fraction (AF) educational level 2	0.33	0.55	0.35	0.09
	Attributable fraction (AF) educational level 1	0.50	0.50	0.44	0.46
	sTAF educational level 3	0.09	0.06	0.09	0.00
	sTAF educational level 2	0.09	0.10	0.14	0.01
	sTAF educational level 1	0.14	0.28	0.08	0.29
	Total attributable fraction (TAF)	0.32	0.43	0.31	0.29
65-80 years					
					
	OR educational level 4	1	1	1	1
	OR educational level 3 (95% CI)	0.7 (0.4-1.6)	0.5 (0.2-1.9)	1.4 (0.7-2.6)	0.7 (0.3-1.6)
	OR educational level 2 (95% CI)	1.2 (0.6-2.3)	0.9 (0.3-2.7)	1.9 (1.1-3.3)	0.6 (0.2-1.7)
	OR educational level 1 (95% CI)	1.4 (0.7-2.6)	1.2 (0.5-2.7)	2.8 (1.6-4.9)	1.0 (0.6-1.8)
	Attributable fraction (AF) educational level 3	-0.25	-0.72	0.21	-0.45
	Attributable fraction (AF) educational level 2	0.12	-0.07	0.37	-0.48
	Attributable fraction (AF) educational level 1	0.20	0.13	0.49	0.02
	sTAF educational level 3	-0.03	-0.03	0.03	-0.04
	sTAF educational level 2	0.04	-0.01	0.14	-0.03
	sTAF educational level 1	0.09	0.11	0.17	0.02
	Total attributable fraction (TAF)	0.10	0.08	0.34	-0.04
All ages					
					
	OR educational level 4	1	1	1	1
	OR educational level 3 (95% CI)	1.8 (1.4-2.2)	1.8 (1.4-2.2)	1.8 (1.4-2.2)	1.1 (0.8-1.4)
	OR educational level 2 (95% CI)	2.1 (1.6-2.6)	2.5 (2.0-3.1)	2.3 (1.9-2.9)	1.4 (1.1-1.7)
	OR educational level 1 (95% CI)	3.0 (2.4-3.7)	2.5 (2.0-3.1)	3.5 (2.8-4.4)	2.5 (2.0-3.0)
	Attributable fraction (AF) educational level 3	0.34	0.39	0.33	0.07
	Attributable fraction (AF) educational level 2	0.40	0.53	0.43	0.23
	Attributable fraction (AF) educational level 1	0.52	0.54	0.54	0.52
	sTAF educational level 3	0.11	0.07	0.09	0.01
	sTAF educational level 2	0.10	0.16	0.14	0.06
	sTAF educational level 1	0.14	0.23	0.13	0.25
	Total attributable fraction (TAF)	0.35	0.46	0.36	0.32

* likelihood expressed as Odds Ratios (OR), where highest educational level (4) is the reference group Weighted data. N = 19 635

Educational level 4 = 15+ years education

Educational level 3 = 13-14 years education

Educational level 2 = 11-12 years education

Educational level 1 = up to 10 years education

Educational inequalities in smoking behaviour was also discernable when analysing the fraction of smoking that could be attributed to lack of higher education (sTAFs and TAFs). In the youngest and middle age groups, Swedish women had higher stratum specific attributable fractions (sTAFs) as well as total attributable fractions (TAFs), indicating a greater impact of educational inequalities on smoking prevalence, compared with Danish women and Swedish and Danish men. The latter groups had almost similar TAFs, although the sTAF for the different educational levels varied slightly between the groups (table 3).

Danish men were the only group among the oldest age group in which education had impact on the smoking. In the other older groups, a lower education even tended to be associated with less smoking, however those associations were not significant.

The gender difference regarding educational impact on daily smoking was greater in Sweden compared to Denmark. In the youngest age group, the TAF for women was 0.55 compared to 0.43 for men. Danish women and men had almost similar TAF, 0.41 and 0.40, respectively (table 2). In the middle age group, TAF was 0.43 for Swedish women, and 0.29 for Swedish men. Again, Danish men and women had almost similar TAF; 0.32 and 0.31, respectively.

Among all "ever smokers" in the youngest and middle age groups, the groups with lower education had significantly increased OR regarding continued smoking (failure to quit), compared to the highest education group (table 4).

**Table 4:** Likelihood* of continued smoking among all ever smokers ("unsuccessful quitters") by educational level, and attributable fractions, for men and women, Denmark and Sweden

		**Women**		**Men**	
		
		**Denmark**	**Sweden**	**Denmark**	**Sweden**
		
18-44 years					
					
	OR educational level 4	1	1	1	1
	OR educational level 3 (95% CI)	1.3(0.9-1.8)	1.6(1.1-2.3)	1.6(1.1-2.3)	1.1(0.8-1.7)
	OR educational level 2 (95% CI)	2.0(1.3-3.1)	1.8(1.3-2.4)	2.1(1.3-3.2)	1.4(1.0-2.0)
	OR educational level 1 (95% CI)	2.4(1.5-3.9)	2.0(1.4-2.8)	2.7 (1.7-4.3)	2.0(1.3-2.9)
	Attributable fraction (AF) educational level 3	0.08	0.19	0.15	0.06
	Attributable fraction (AF) educational level 2	0.20	0.22	0.20	0.15
	Attributable fraction (AF) educational level 1	0.24	0.25	0.25	0.25
	sTAF educational level 3	0.04	0.04	0.05	0.01
	sTAF educational level 2	0.04	0.10	0.05	0.07
	sTAF educational level 1	0.05	0.05	0.06	0.06
	Total attributable fraction (TAF)	0.12	0.19	0.16	0.14
45-64 years					
					
	OR educational level 4				
	OR educational level 3 (95% CI)	1.9 (1.3-2.9)	1.2 (0.8-1.7)	1.9 (1.3-3.0)	0.8 (0.6-1.2)
	OR educational level 2 (95% CI)	2.1 (1.4-3.2)	1.9 (1.3-2.8)	1.8 (1.2-2.6)	0.9 (0.7-1.4)
	OR educational level 1 (95% CI)	4.6 (2.8-7.7)	1.8 (1.4-2.5)	2.3 (1.4-3.6)	1.6(1.2-2.1)
	Attributable fraction (AF) educational level 3	0.27	0.08	0.26	-0.16
	Attributable fraction (AF) educational level 2	0.29	0.29	0.23	-0.03
	Attributable fraction (AF) educational level 1	0.44	0.28	0.30	0.23
	sTAF educational level 3	0.08	0.01	0.07	-0.02
	sTAF educational level 2	0.08	0.05	0.09	-0.00
	sTAF educational level 1	0.12	0.15	0.06	0.14
	Total attributable fraction (TAF)	0.28	0.21	0.21	0.11
65+ years					
					
	OR educational level 4	1	1	1	1
	OR educational level 3 (95% CI)	0.7 (0.3-1.5)	0.4 (0.1-1.5)	0.9 (0.4-1.7)	0.6 (0.3-1.4)
	OR educational level 2 (95% CI)	1.0 (0.5-2.2)	1.0 (0.3-3.0)	1.1 (0.7-2.0)	0.9 (0.3-2.4)
	OR educational level 1 (95% CI)	1.6 (0.8-3.2)	1.4 (0.6-3.3)	1.7 (0.9-3.0)	1.1 (0.6-2.0)
	Attributable fraction (AF) educational level 3	-0.26	-0.86	-0.10	-0.49
	Attributable fraction (AF) educational level 2	0.02	0.01	0.07	-0.10
	Attributable fraction (AF) educational level 1	0.19	0.21	0.24	0.07
	sTAF educational level 3	-0.04	-0.04	-0.01	-0.04
	sTAF educational level 2	0.01	0.00	0.03	-0.01
	sTAF educational level 1	0.09	0.17	0.08	0.05
	Total attributable fraction (TAF)	0.06	0.13	0.10	0.01
All ages					
					
	OR educational level 4	1	1	1	1
	OR educational level 3 (95% CI)	1.4 (1.1-1.8)	1.4 (1.1-1.7)	1.7 (1.3-2.2)	1.0 (0.8-1.2)
	OR educational level 2 (95% CI)	1.5 (1.2-2.0)	2.0 (1.6-2.5)	1.5 (1.1-1.9)	1.6 (1.3-2.0)
	OR educational level 1 (95% CI)	2.3 (1.7-3.1)	1.5 (1.2-1.9)	2.1 (1.6-2.7)	1.2 (1.0-1.5)
	Attributable fraction (AF) educational level 3	0.14	0.15	0.20	-0.02
	Attributable fraction (AF) educational level 2	0.17	0.28	0.15	0.23
	Attributable fraction (AF) educational level 1	0.28	0.19	0.25	0.11
	sTAF educational level 3	0.05	0.03	0.06	0.00
	sTAF educational level 2	0.04	0.09	0.05	0.07
	sTAF educational level 1	0.07	0.08	0.06	0.05
	Total attributable fraction (TAF)	15.8	18.8	16.2	11.0

* likelihood expressed as Odds Ratios (OR), where highest educational level (4) is the reference group Weighted data. N = 19 635

Educational level 4 = 15+ years education

Educational level 3 = 13-14 years education

Educational level 2 = 11-12 years education

Educational level 1 = up to 10 years education

OR's and AFs were highest in the lowest education group (level 1). An exception was among Swedish middle aged men, among which only the lowest education group but not educational level 2 and 3 had significantly increased OR regarding continued smoking, compared to the highest education group (level 4).

The gender differences in the youngest age group were opposite in Sweden and Denmark. In the Swedish population, the women had higher TAF for current smoking than the men, while in the Danish population the men had higher TAF than the women. In the middle age group, however, both Swedish and Danish women had higher TAF than their male counterparts.

In the oldest age group, no significant association between current smoking and education was found.

## Discussion

The main finding of our study is that the socioeconomic patterning of smoking, based on level of education and expressed as the relative contribution to the total burden of smoking exposure, is rather different in Sweden and Denmark. Moreover, these differences are modified by gender and age. As a general pattern, socioeconomic differences in Sweden tend to contribute more to the total burden of this habit among women, especially in the younger age groups. Regarding men, the patterns were much more similar between the two countries. When we analysed the same type of patterns regarding continued smoking/unsuccessful quitting, which ought to be a more sensitive measure of recent tobacco policies, they were similar for women, but somewhat different for men. Here we found that socioeconomic differences contributed more to overall continued smoking in Danish men, especially in the middle-age and older age strata. This seems to confirm our hypothesis that the specific interplay between socioeconomic development and tobacco policy on the national level, in fact do produce different socioeconomic patterns in smoking in countries that are considered to be similar in many ways, and also have a similar situation regarding overall health inequity.

Our results also confirm previous findings of markedly higher prevalence of smoking in Denmark compared to Sweden. Also the prevalence of "ever smoking" (i.e. those who ever had started to smoke, regardless whether they had quit or continued to smoke) was considerably higher in Denmark compared to Sweden. The educational inequalities in smoking habits were clear in both countries, among those younger than 65 years. The AFs were considerable for the younger and middle aged groups, indicating a substantial proportion of smoking in both countries that would be prevented if the low educated did smoke at a similar rate as the high educated. The highest AF's were found in the youngest age group, which implies that inequality in smoking is increasing over time in both countries.

The TAF measure should be considered as the share of smoking that could be avoided, given that the low educated smoked to a similar extent as the high educated. The TAF may therefore be regarded as the preventive potential or need for change in striving for an equal level of smoking across all education levels (i.e. equal to that of the group with the highest level of education). The advantage of this measure is that it takes into consideration the differing size of the educational level groups in the compared countries and in the three compared age groups. Furthermore, it help us to avoid the problem with different general levels of a risk factor (here smoking) in compared populations, which could be misleading in the sense that the same absolute (prevalence) differences inevitably will come out as different relative measures (e.g. rate ratios or odds ratios) if the general level of the studied risk factor differs between the compared populations. For example, a high population prevalence of smoking in Denmark will yield lower relative estimates of inequality given the same absolute differences between educational strata as in Sweden, where the population prevalence is considerably lower [14].

The highest TAF, i. e., the total proportion of smoking in the population that would be prevented if the low educated smoked as much or, or as little as, the highest educated, was found for Swedish young women. This could be a sign that the educational inequalities in smoking in Sweden will increase in the future, which is in accordance with previous studies indicating this disheartening vision of the future [6,7,15]. A previous study analysing BMI change over five years in the Swedish part of the present study population showed that low education among especially young women was associated with a higher BMI increase, accentuating the possibility that this group could be under risk of increasing ill health in the future [16].

In the oldest age group, low education was not at all associated with more smoking, at least not in Sweden. This may partly depend on a selection effect with a higher mortality among exposed cases, that is, low educated smokers. It is noteworthy that it is much less common with the highest educational level in the oldest groups. A high education was not as necessary a few decades ago as it is today to achieve high position jobs and a higher socio economic status. Hence, education may be an indicator of different phenomena in the oldest age group, compared with the other age groups.

In the total population, 46% of the smoking among Swedish women could be attributed to low education. This is much higher than the 32% of the male smoking in Sweden that could be attributed to low education. The educational inequalities were smaller in Denmark compared with among Swedish women, and in Denmark there were almost no gender difference; the TAF was 0.35 and 0.36 for women and men, respectively. One possible explanation of the difference between Swedish and Danish men could be the rather frequent use of snuff (oral use of tobacco) among Swedish men. Snuff is almost exclusively used in Sweden, where the prevalence of users equal the level of the smoking prevalence among men, but only reach a tenth of that level among women while this habit is negligible in Denmark in both sexes. Moreover, use of snuff is more than twice as common among low educated men than among high educated men [17]. The counterfactual situation where snuff did not exist in Sweden would therefore most likely increase educational inequality in smoking, especially continued smoking/unsuccessful quitting, among Swedish men. This could indicate that in the absence of snuff, socioeconomic differences might contribute more to the total burden of smoking in both sexes in Sweden, compared to Denmark.

In a recent paper comparing health equity policies in Sweden and Denmark, Vallgårda [18] observes some striking differences between the two countries: Health inequalities was put on the policy agenda in the early 1980-ies in Sweden and it was already from the beginning the *gradient *which was put in focus, whereas in Denmark the issue was raised about ten years later and mainly became a matter of addressing poor health in marginalized groups. Moreover, in Sweden health inequalities were put into a broader focus including both behaviour and living conditions, while they in Denmark mainly were regarded as unhealthy behaviours among the poor, according to Vallgårda. The uplift of tobacco control measures in Sweden began in 1963 when the Swedish Government appointed an expert panel within the Swedish Health Care and Medical Services Department (the predecessor of the National Board on Health and Welfare). This group was assigned to investigate the relationship between tobacco use and its adverse effect on health. In 1964, the group was given a yearly budget of half a million SEK/year. The final report of this group submitted to the government 1974 became as a break through for a new national tobacco preventive policy. Since then a systematic and long term political targets and legislations on how to reduce tobacco consumption in Sweden were consecutively implemented [19]. Hence, the Swedish tobacco control policy was characterized by a high level of taxation, advertising ban, indoor work and public place legislations, age restriction on sale of tobacco products to minors and support for smoking cessation [10], while Danish tobacco control policies lag behind both in terms of legislation and implementation almost in all aforementioned areas [20]. However, other contemporary determinants such as public debates, liberal views on smoking and other substance abuse predictably constrained the development of the tobacco control policies in Denmark.

Our results might thus imply an overall picture, where the interplay between the socioeconomic development and specific traits in national tobacco policies have produced the observed the patterns of socioeconomic differences in smoking. This interplay could be expected to be rather intricate in its detailed mechanisms, which also may shift over time, and therefore could not be exhaustively analysed by means of the data in this particular study. A "default hypothesis" could be that it might be the intensity of anti-smoking policies and the implementation of these that produce the observed results. That is; the harder one tries, the better overall results in decreasing the population prevalence in smoking, but at the expense of increasing inequality in the burden of smoking in the same population. If this were true, the results of our study call for increasing the efforts to develop policies and intervention strategies which could break this trend of inverse relation between overall efficiency and efficiency in decreasing inequity.

In the present study, we used education as a measure of socioeconomic status. Various measures of socioeconomic status, income and education have been used in previous studies examining social inequalities in health or health related behaviour, and smoking has been found to be associated with structural, material as well as perceived dimensions of socioeconomic disadvantage [21,22]. A previous study compared education and income to determine which was most strongly related to smoking, and whether each factor had an independent effect [7]. The results showed that education was a strong predictor of smoking in Europe, and educational inequalities were larger than income related inequalities among both men and women in northern Europe. The independent effect of education on smoking was larger than the independent effect of income, although also the independent effect of income was statistically significant in some countries. The educational inequalities were largest among age groups younger than 44 years, which is in accordance with our findings.

The test-retest reliability of smoking history has been showed to be relatively high, although male gender, and lower educational level was associated with lower reliability [23].

The survey methods differed between the Danish and the Swedish part of the data, since personal interviews were performed in Denmark and a mailed self-administered questionnaire was used in Sweden. This will probably explain the observed difference of 12% in the participation rate between the two countries. Usually it is individuals with low education and adverse health behaviours who are most easily lost in surveys of this type, which could imply that a higher proportion of those were included in the Danish data, thus introducing a selection bias which could lead to an underestimation of the smoking prevalence as well as the observed inequalities in the Swedish data. However, non-desirable behaviours tend to be underreported in personal interviews compared to self-administered questionnaires, which might have counteracted part of that possible bias.

In the analysis of the data set used in this study, we had the opportunity to not only compare a "snapshot" of inequality in tobacco smoking in one country, but we also had the opportunity to contrast this picture with the same type of picture from another country, and to a reasonable extent by means of comparing three age strata, in addition get an impression of the time dimension.

Thus we have the "vertical" dimension (i.e. inequalities in tobacco smoking), the "horizontal" dimension (country/policy level) and the dimension of time, which we can use for our overall analysis.

Trying to focus on the overall picture resulting from our analysis, the main findings can perhaps be expressed in two conclusive statements, somewhat surprisingly compatible with each other: Firstly, the best educated individuals seemed to have benefited the most from the assumingly predominant population strategy in Sweden. However, the lowest educated group in Sweden seem to have decreased their smoking prevalence more than the corresponding group in Denmark, although the gap increased compared with the highest educated group. The predominant population approach in Sweden thus led to a more beneficial effect in terms reduced tobacco smoking, both in the lowest educational group *and *in the highest, although inequality in smoking increased. Vice versa, the predominant risk group approach in Denmark have in a comparative perspective between Sweden and Denmark, been to the disadvantage for *both *the highest and the lowest educational groups, although the inequality in smoking, somewhat paradoxically, seem to have decreased in Denmark.

## Conclusion

The results imply that Swedish anti-smoking policy and/or implemented measures have been less effective in a health equity perspective among the younger generation of women, but more effective among men, compared to Danish policy implementation. The results also raises the more general issue regarding the possible need for a trade-off principle between overall population efficacy versus equity efficacy of anti-tobacco, as well as general public health policies and intervention strategies.

## Abbreviations

AF: Attributable fraction; OR: Odds Ratio; S-TAF: stratum specific total attributable fraction; TAF: Overall Total attributable faction

## Competing interests

The authors declare that they have no competing interests.

## Authors' contributions

POÖ was the PI and main applicant and FD and NKR were co-invetsigators and co-applicants of the grant for this study from the Swedish Council for Working Life and Social Research (FAS 2004-1075). POÖ and FE drafted the manuscript, and FE performed the main writing and statistical analyses. MG contributed with discussions regarding the statistical methods. POÖ, FD, NR, IA, KM have all contributed to the design of this study, interpretation of data and revision of the manuscript. All authors read and approved of the final manuscript.

## Pre-publication history

The pre-publication history for this paper can be accessed here:

http://www.biomedcentral.com/1471-2458/10/9/prepub
